# Expression and immunoassay application of full-length recombinant antibody recognizing chlorantraniliprole using *Pichia pastoris* expression system

**DOI:** 10.1007/s44297-026-00085-4

**Published:** 2026-08-24

**Authors:** Lianrun Huang, Panpan Cui, Yang Zhang, Yanling Wu, Yuan Ding, Maojun Jin, Xiude Hua

**Affiliations:** 1 College of Plant Protection, Nanjing Agricultural University, Nanjing, China; 2 State & Local Joint Engineering Research Center of Green Pesticide Invention and Application, Nanjing, China; 3 Institute of Quality Standards & Testing Technology for Agro-Products, Chinese Academy of Agricultural Sciences, Beijing, China

**Keywords:** Recombinant antibody, Yeast expression system, Immunoassay, Chlorantraniliprole

## Abstract

Full-length recombinant antibodies (rAbs) expressed in yeast offer the advantage of a eukaryotic system ensuring proper folding and bioactivity, along with high yield and low cost. In this study, plasmids encoding the light chain and heavy chain of the anti-chlorantraniliprole monoclonal antibody (mAb) were constructed under the control of inducible alcohol oxidase promoters and equipped with secretory signal peptides. Subsequently, the rAb was expressed in *Pichia pastoris* and further utilized to develop a lateral flow immunoassay (LFIA). The visual limit of detection (vLOD) of the rAb-based LFIA was 0.63 ng/mL, which was twofold lower than that of the mAb-based LFIA. Validated via recovery experiments, the rAb-LFIA demonstrated that the vLOD of chlorantraniliprole in rice, apple, and grape samples complied with the maximum residue limits specified in Chinese and European Union food safety standards. Moreover, the rAb-LFIA exhibited reliability comparable to that of UPLC-MS/MS in the detection of blind samples.

**Supplementary Information:**

The online version contains supplementary material available at 10.1007/s44297-026-00085-4.

## Introduction

In recent years, immunoassays have emerged as a prevalent analytical technique for the detection of agricultural contaminants and toxins, owing to their simple operation, low cost, high sensitivity, and high throughput [1, 2]. The cornerstone of immunoassays is high-quality antibodies [3]. At present, monoclonal antibodies (mAbs) are the most commonly used antibodies in immunoassays. Traditionally, mAbs have been predominantly generated via hybridoma technology. However, hybridoma cells have genetic instability, making them prone to antibody gene mutations or loss [4]. Additionally, the production of antibodies relies on substantial quantities of laboratory animals, which incurs high costs and raises ethical concerns regarding animal welfare [5, 6]. These limitations hinder mAbs application in standardized reagent development.

The emergence of recombinant antibody (rAbs) technology provides an effective way to address these issues. Through genetic engineering, the variable-region genes of antibodies can be cloned and inserted into expression vectors to generate recombinant antibodies in appropriate expression systems [7]. This strategy ensures the fidelity of antibody sequences and batch-to-batch consistency in product quality [8]. Additionally, it delivers lower manufacturing costs, decreased reliance on experimental animals, and greater flexibility for targeted antibody engineering [9, 10]. The recombinant forms of mAb can be classified into two types based on their molecular structures: full-length antibodies and antibody fragments [11]. Compared with antibody fragments, full-length antibodies retain the native structural conformation and antigen-binding affinity of the parental antibody to a greater extent, thereby exhibiting superior practical applicability [12].

The expression systems for recombinant antibodies are diverse, including prokaryotic systems (such as *Escherichia coli*), lower eukaryotic systems (such as yeast), and higher eukaryotic systems (such as mammalian cells) [13, 14]. The *Escherichia coli* system has the advantages of fast growth, low cost, and simple genetic manipulation. It lacks post-translational modification (PTM) mechanisms and secretion pathways [15, 16]. These factors make it usually only suitable for producing antibody fragments, such as fragment antigen-binding (Fab) and single-chain variable fragment (scFv) [17]. Mammalian cells, including CHO and HEK293 cells, are capable of performing complex post-translational modifications such as human-like glycosylation, rendering them the gold standard for producing therapeutic full-length antibodies [18]. However, the long culture cycle, high cost, and complexity of operation of mammalian cell culture make them not fully suitable for the economical large-scale production of antibodies [14].

As unicellular eukaryotes, the yeast expression system integrates the merits of prokaryotic platforms, including rapid proliferation, low cultivation costs, and facile large-scale fermentation, as well as the eukaryotic capacities of secretory pathways and complete post-translational modification machinery [19, 20]. The difference in glycosylation modifications between mammalian cells and yeast cells must be considered, as it can affect the structure and activity of rAbs [21]. The glycosylation of the Fc region in the therapeutic antibody significantly affects the Fc-mediated effector functions of the antibody [22]. For the antibodies used in in vitro diagnosis, the impact of glycosylation is smaller. Das et al. [23] constructed a yeast-based full-length recombinant tocilizumab expression system without glycoengineering and demonstrated that its binding affinity for IL6R was comparable to that of commercial tocilizumab. The methylotrophic yeast *Pichia pastoris*, known for its potent methanol-inducible alcohol oxidase promoter (AOX1), high-density fermentation capability, and efficient protein secretion ability, has emerged as a mature platform for producing various heterologous proteins [24]. These characteristics satisfy the requirements for large-scale, stable, and cost-effective antibodies applicable to pesticide residue immunoassays. To the best of our knowledge, no yeast-based expression system has currently been established for the production of recombinant full-length antibodies targeting pesticides.

In this study, chlorantraniliprole (CAP), a widely applied anthranilic diamide insecticide with potential hazards to human health [25], was employed as the target analyte. First, two yeast expression vectors were constructed to separately encode the light and heavy chains of full-length recombinant antibodies. The *Pichia pastoris* system was utilized for the expression of recombinant full-length CAP antibody, with key expression parameters systematically optimized. Subsequently, a lateral flow immunoassay (LFIA) was established based on the rAbs for the detection of CAP in agricultural samples, thereby verifying the practical applicability of the prepared antibodies. In general, this article presents a promising alternative for the stable, economical, and scalable production of antibodies for immunoassays and provides an effective reagent for the rapid detection of CAP residues.

## Results

### Construction of CAP expression plasmids

**Fig. 1:**
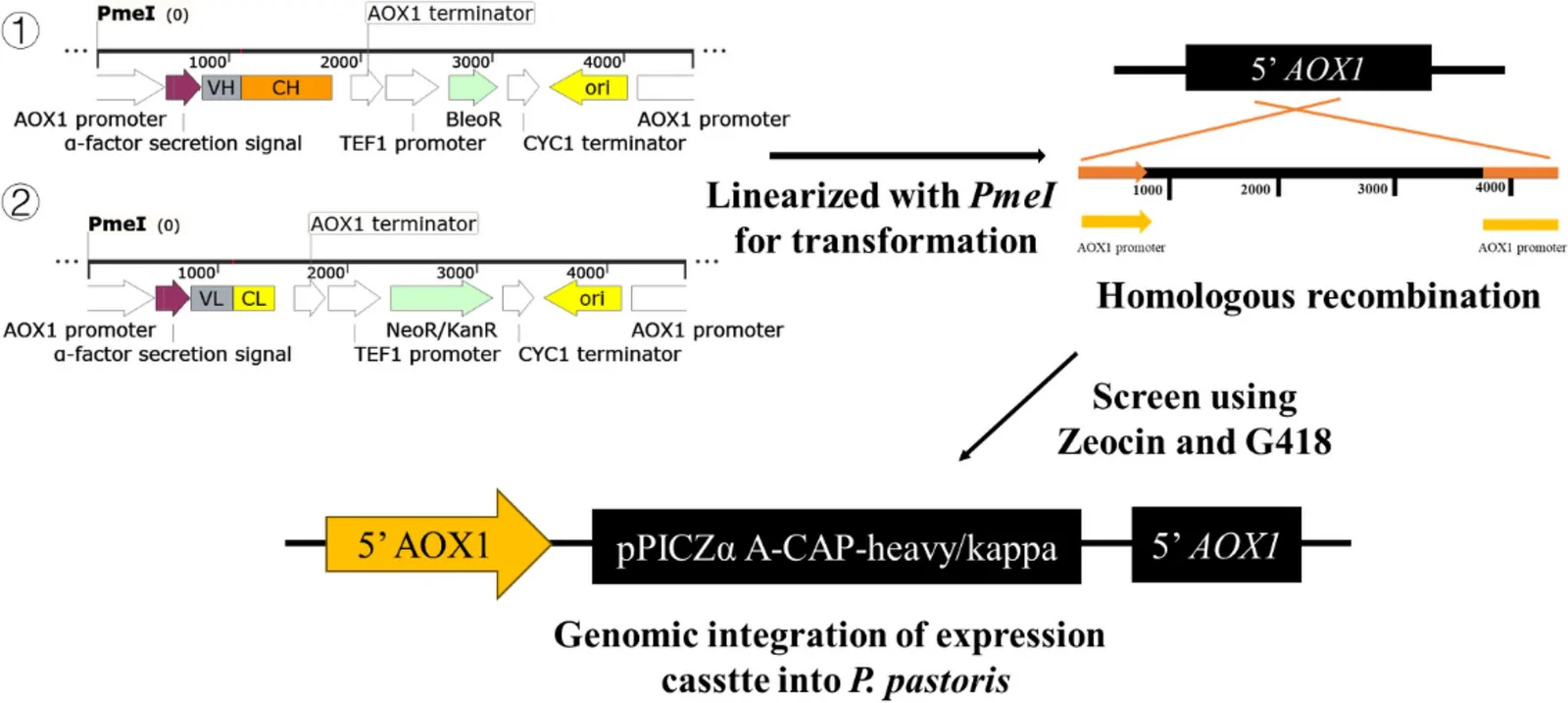
Construction process of the full-length rAb expression system

**Fig. 2:**
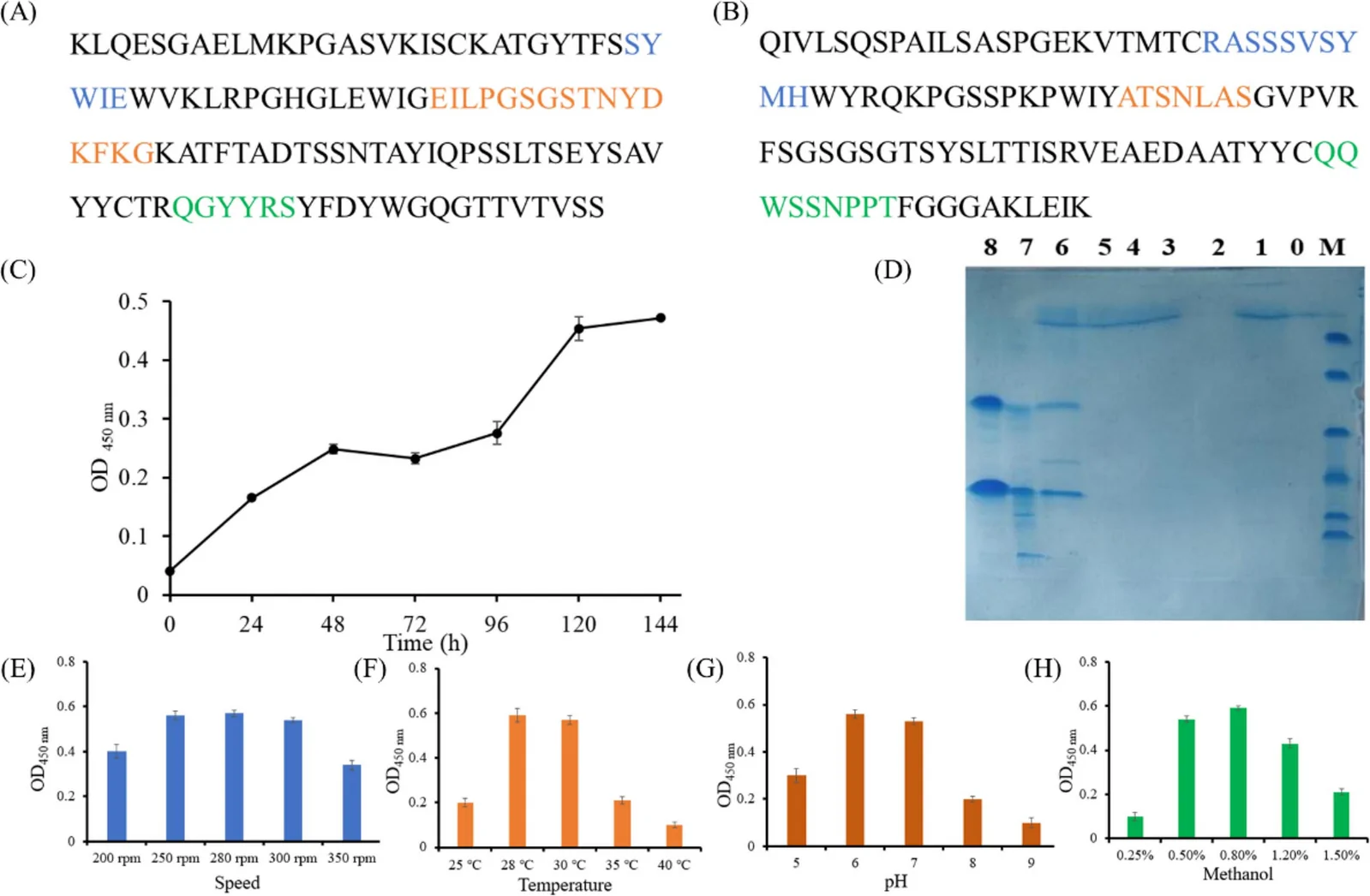
Sequence characterization of variable regions from mAb 5C5B9 and expression optimization of its derived recombinant antibody. **A**, **B** Deduced amino acid sequences of VH (**A**) and VL (**B**) of mAb 5C5B9. CDR1, CDR2 and CDR3 are highlighted in blue, orange and green, respectively. **C** Accumulation kinetics of the recombinant antibody in the culture supernatant. Antibody levels were determined by indirect ELISA (*n* = 3). **D** SDS-PAGE analysis of antibody expression in culture supernatants at different induction time points. Lane M: low-molecular-weight protein marker; Lanes 0–7: culture supernatants harvested at 0, 24, 48, 72, 96, 120 and 144 h post-induction, respectively; Lane 8: purified parental mAb 5C5B9 as a positive control. **E**–**H** Effects of key culture parameters on recombinant antibody expression levels (quantified by indirect ELISA at OD_450 nm_): **E** shaking speed, (**F**) induction temperature, **G** initial medium pH, and **H** methanol concentration

The construction process of the plasmids is shown in Fig. 1. The isotype of mAb 5C5B9 was determined as IgG1 (heavy chain) and kappa (light chain) by using an Isotyping Kit for Mouse Monoclonal Antibody (Sino Biological Inc., Beijing, China). The extracted mRNA was validated by 1% agarose gel electrophoresis. As shown in Fig. S1A, the three RNA bands on the gel did not show any smearing or trailing, with band sizes of 28S, 18S, and 5S from top to bottom. After reverse transcription and polymerase chain reaction (PCR) amplification, it can be observed from the PCR gel electrophoresis image that the variable regions of heavy chain (VH) and light chain (VL) gene fragments are approximately 300–400 bp in size (Fig. S1B), consistent with the theoretical values. After Sanger sequencing, the gene sequences of the PCR-amplified fragments were analyzed on the IMGT website. The amino acid sequences and complementarity determining regions (CDRs) region sequences of the VH and VL of the antibody are shown in Fig. 2A and B. These sequences were input into the glycosylation site prediction tools NetOGlyc and NetNGlyc [26, 27]. The results indicated that there were no common glycosylation sites in the variable region, suggesting that the probability of glycosylation causing the loss of activity of rAbs is relatively low.

The gel electrophoresis bands for constant regions of heavy chain (CH) and light chain (CL) gene fragments were close to 940 bp and 340 bp, matching the theoretical expectations (Fig. S1C). The antibody constant region genes and the pPICZα A vector were integrated through enzyme digestion and enzyme-linked reaction (Fig. S1D). Finally, the gene fragments of VH and VL were inserted into pPICZα A-heavy and pPICZα A-kappa to construct pPICZα A-CAP-heavy and pPICZα A-CAP-kappa (Fig. S1E and F). The results of Sanger sequencing and gel electrophoresis indicate successful synthesis of the plasmid.

### Construction and cultivation of the expression strain

The colony PCR results indicate that the two plasmids were successfully integrated into the yeast genome (Fig. S2). In the expression cassette of the CAP rAb, the heavy chain and kappa chain genes are controlled by the AOX1 promoter and connected to the α-mating factor (α-MF) secretion signal sequence for extracellular expression. Genomic integration was achieved by linearizing the expression vector at the SacI site within the AOX1 promoter, which exposes the flanking homology sequences of the linearized plasmids. The linearized plasmids were subsequently integrated into the genomic AOX1 promoter locus through yeast homologous recombination machinery.

To achieve the optimal expression level, the cultivation time and conditions were optimized. First, the expression levels of rAb at different cultivation times were determined by enzyme-linked immunosorbent assay (ELISA) and gel electrophoresis. As shown in Fig. 2C and D, the rAb content in the supernatant increased with cultivation time, but there were more impurities in the supernatant after 144 h. Therefore, the cultivation time was set at 120 h. By altering the temperature, rotational speed, pH, and methanol content during induction of the expression system, the binding activity of the recombinant antibody to the antigen in the supernatant was determined through ELISA, further establishing the optimal expression conditions. As shown in Fig. 2E to H, the optimal conditions for expression were at 28 °C with a shaker speed of 280 rpm, using a culture medium with pH 7 and containing 0.8% methanol for expression.

### Recombinant antibodies expressed in yeast retain the specificity of the parent antibody and exhibit high yield

**Fig. 3:**
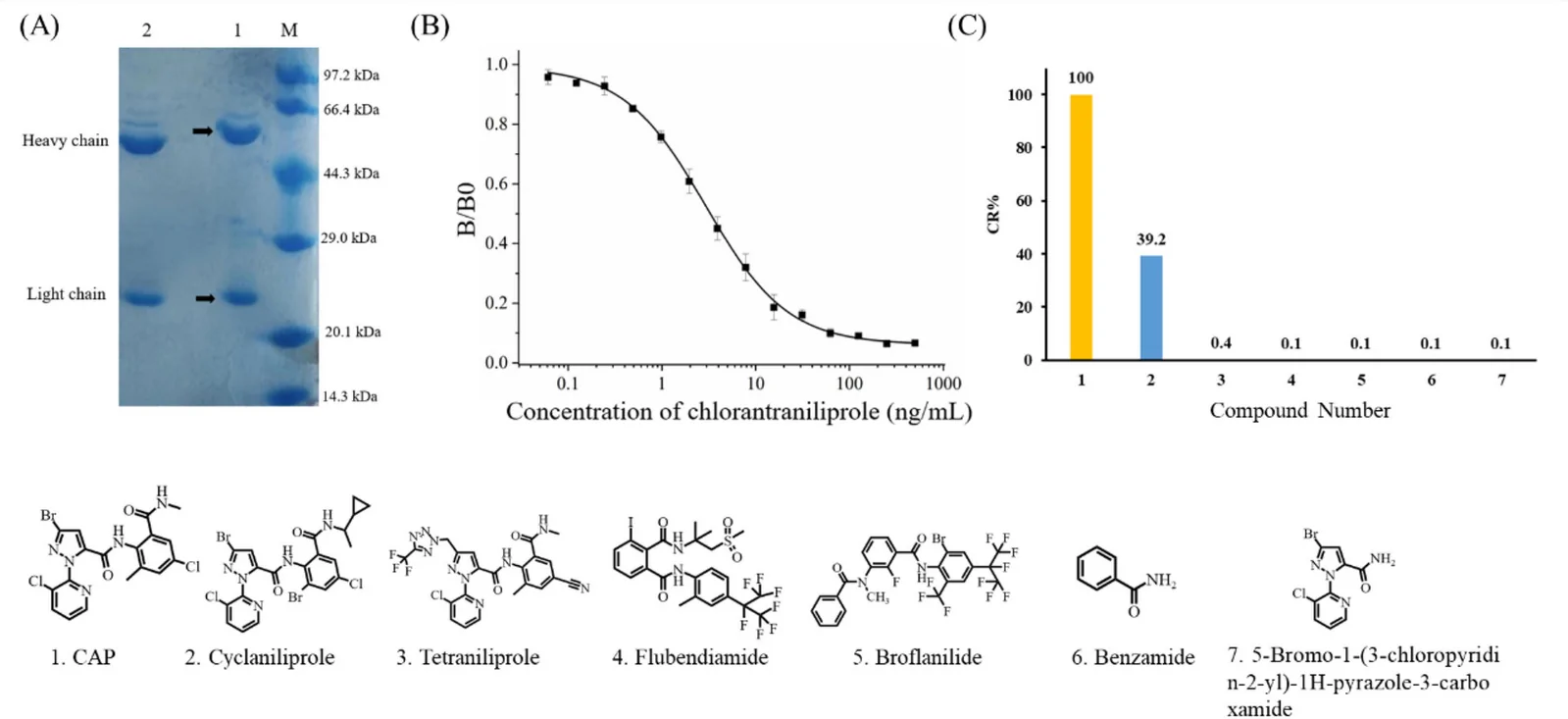
Purity verification, sensitivity determination and specificity analysis of the purified rAb. **A** Purity assessment of the purified recombinant antibody via SDS-PAGE under reducing conditions. Lane M: low-molecular-weight protein marker; Lane 1: purified rAb; Lane 2: parental monoclonal antibody 5C5B9. Arrows indicate the bands corresponding to the heavy chain and light chain. **B** Sensitivity determination of the rAb against CAP via ic-ELISA (*n* = 3). **C** Specificity analysis of the rAb. CR% toward CAP and its structural analogs were calculated based on ic-ELISA results

Following the best expression time and conditions for rAb, the supernatant was collected and purified using Protein A. The purified recombinant antibody was identified by 10% sodium dodecyl sulfate‒polyacrylamide gel electrophoresis (SDS‒PAGE). Under reducing conditions, protein bands appeared at 57 kDa and 25 kDa (Fig. 3A), corresponding to the sizes of the antibody heavy chain and kappa chain, respectively.

After determining the optimal amounts of rAb and coated antigen using a checkboard titration [28], the sensitivity of the antibody was then measured using an indirect competitive ELISA (ic-ELISA) [29]. As shown in Fig. 3B, the half maximal inhibitory concentration (IC_50_) of ic-ELISA based on the rAb was 2.9 ng/mL, with a linear range (IC_10_-IC_90_) of 0.4–25.8 ng/mL and a limit of detection (LOD) of 0.4 ng/mL. Compared to the parent mAb 5C5B9 (IC_50_ is 0.24 ng/mL), the sensitivity has decreased by ten times, but it still retains an acceptable level of sensitivity.

The IC_50_ values of the ic-ELISA for CAP analogs (cyclaniliprole, tetraniliprole, flubendiamide, broflanilide, and benzamide) were determined (Fig. 3C). The CR values for cyclaniliprole and tetraniliprole were 39.2% and 0.4%, respectively. The cross-reactivity (CR) values for the other analogs were below 0.1%. The results show that the specificity of the rAb is the same as that of the parental mAb [30]. The results above indicate that the rAb expressed by *Pichia pastoris* retained the sensitivity and specificity of the parent mAb.

The rAb was successfully expressed in *Pichia pastoris* at 9.3 mg/L (n = 3), consistent with typical full-length IgG yields (5–50 mg/L) in *Pichia pastoris* shake flask cultures [31]. For comparison, lab-scale hybridoma cultures yield 10–100 mg/L [32], transient HEK293F expression produces 50–350 mg/L [33], and industrial CHO fed-batch processes achieve 1–10 g/L [34]. From a cost perspective, *Pichia pastoris* shake flask cultures ($80–300/mg) are more economical than lab-scale mammalian systems (hybridoma: $100–500/mg; HEK293F: $200–800/mg) [35, 36], while high-density *Pichia pastoris* fermentations provide a competitive mid-scale alternative ($10–50/g) to industrial CHO production ($0.5–5/g) [35, 37].

### Determination of the optimal buffer solution for LFIA

**Fig. 4:**
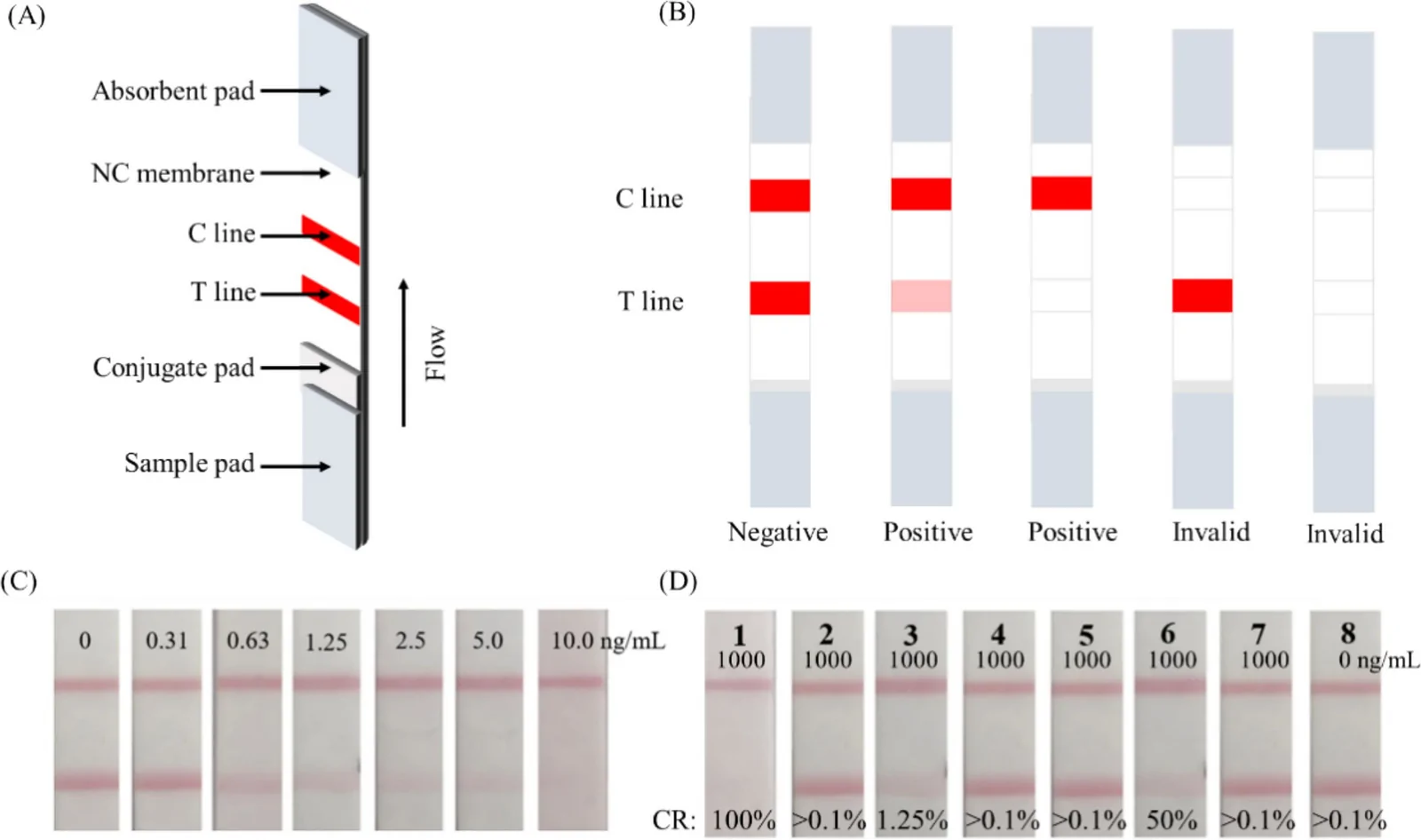
Schematic structure, result interpretation and analytical performance of the rAb-based LFIA strip. **A** Schematic diagram of the LFIA strip composition. The strip is assembled with a sample pad, conjugate pad, NC membrane and absorbent pad from bottom to top. The control (**C**) line and test (T) line are coated on the NC membrane, and the arrow indicates the direction of sample capillary flow. **B** Schematic illustration of LFIA result judgment criteria, showing representative patterns of negative, positive and invalid outcomes. **C** Visual sensitivity of the LFIA for CAP detection. Strips were tested with CAP standard solutions at serial concentrations of 0, 0.31, 0.63, 1.25, 2.5, 5.0 and 10.0 ng/mL. **D** Specificity evaluation of the LFIA via a cross-reactivity assay. Strips 1–7 were tested with 1000 ng/mL of each CAP structural analog, and strip 8 served as the blank control with working buffer. Compounds: 1, CAP; 2, broflanilide; 3, tetraniliprole; 4, 5-bromo-1-(3-chloropyridin-2-yl)−1H-pyrazole-3-carboxamide; 5, flubendiamide; 6, cyclaniliprole; 7, benzamide

The assembly and interpretation of LFIA are shown in Fig. 4A and B. To further improve the sensitivity of LFIA, optimization was carried out on the sodium ion concentration, pH value, surfactant, and methanol content in the buffer solution (Fig. S3). When the sodium ion concentration is greater than 0.14 mol/L, both the color of the test (T) line and control (C) line will appear lighter, making it difficult to observe the test results. If the sodium ion concentration is below 0.14 mol/L, the sensitivity of the LFIA decreases. Hence, the concentration of sodium ions was set at 0.14 mol/L. Similarly, the optimal pH value and tween-20 concentration were determined to be 7.4 and 0.05%, respectively. Methanol can enhance the solubility of analytes in buffer solution, but excessive methanol can affect antigen–antibody binding. When the methanol content exceeds 2.5%, the sensitivity of the test strip significantly decreases. Therefore, the optimal methanol content was 2.5%. In conclusion, the best working buffer is phosphate buffer (pH 7.4) containing 0.14 mol/L Na^+^, 0.05% tween-20 and 2.5% methanol.

### LFIA based on rAb has excellent sensitivity and good specificity

Under the optimal conditions, the sensitivity of the LFIA based on rAb was determined using a series of CAP standard solutions (0, 0.31, 0.63, 1.25, 2.5, 5.0 ng/mL). The test result was positive when the CAP concentration was greater than or equal to 0.63 ng/mL, whereas it was negative at concentrations below this threshold (Fig. 4C). Therefore, the visual LOD (vLOD) of the LFIA was 0.63 ng/mL. Compared with the LFIA reported by Xu et al. [38], the rAb-based LFIA offered fourfold enhanced sensitivity. Compared with the ELISA method [39, 40], it shortened the detection time by over tenfold, while its sensitivity was enhanced 2.6-fold and 2.4-fold, respectively (Table 1).

**Table 1:** Comparison of the rAb-based LFIA with mAb-based immunoassay methods

Immunoassay	Sensitivity (ng/mL)	Detection time (min)	Reference
ELISA(mAb)	1.6	135	[39]
ELISA(mAb)	1.5	180	[40]
LFIA(mAb)	2.5	15	[38]
LFIA(5C5B9)	1.25	10	[30]
LFIA (rAb)	0.63	10	This study

The standard solutions (1000 ng/mL) of cyclaniliprole, tetraniliprole, flubendiamide, broflanilide, and benzamide were detected by LFIA. As shown in Fig. 4D, the CR of cyclaniliprole and tetraniliprole were determined to be 50% and 1.25% using the previous method [30], respectively, while all other structural analogs did not exhibit any cross-reactivity. Compared with the AuNP-LFIA established using parental antibodies [30], the LFIA based on rAb retained sensitivity and specificity while cutting the overall manufacturing cost of the assay.

### LFIA based on rAb can meet detection requirements for CAP in various agricultural product matrices

Sample matrix effects can negatively affect the accuracy and sensitivity of immunoassays. In most cases, the matrix effect can be eliminated by dilution [41]. As shown in Fig. S4, 48-fold dilution of rice, apple, sweet potato, and grape sample extracts (twofold extraction followed by an additional 24-fold dilution) yielded a detection sensitivity equivalent to that observed in matrix-free buffer. This result indicated that the matrix effect can be eliminated after a 48-fold dilution for the samples of rice, apple, sweet potato, and grape.

After dilution, the extraction solutions of spiked samples were detected by LFIA. As shown in Table 2, the vLODs of LFIA for CAP in apple, sweet potato, grape, and rice samples were 0.04 mg/kg. According to EU regulations (EU 2022/1343), the maximum residue limits (MRLs) of CAP in brown rice, apples, sweet potatoes, and grapes are 0.4, 0.4, 0.02, and 1.0 mg/kg, respectively. In China, the MRLs of CAP in rice, apples, sweet potatoes, and grapes are set at 0.5, 2, 0.02, and 1.0 mg/kg, respectively (GB 2763–2026). The vLODs of LFIA for CAP in rice, apples, and grapes all satisfy the MRL criteria specified in both the Chinese standard and the EU regulation. The vLOD of LFIA for CAP in sweet potatoes was 0.04 mg/kg, which exceeds the statutory MRLs established by both Chinese and EU regulatory standards. This limitation renders the assay insufficient for the accurate detection of CAP residues in sweet potatoes, indicating a need for further improvement in detection sensitivity.

**Table 2:** Results of chlorantraniliprole residue detection by the LFIA on spiked samples (*n* = 3)

Sample	Spiked (mg/kg)	Results of LFIA	Sample	Spiked (mg/kg)	Results of LFIA
Brown rice	0		Sweet potato	0	
	0.02			0.02	
	0.04			0.04	
	0.08			0.08	
	0.16			0.16	
Apple	0		Grape	0	
	0.02			0.02	
	0.04			0.04	
	0.08			0.08	
	0.16			0.16	

Note: (+): positive; (±) weakly positive; (−): negative

### The results of LFIA are consistent with those of UPLC-MS/MS

Ten samples of rice spiked with an unknown concentration of CAP were simultaneously detected using LFIA and Ultra-high performance liquid chromatography-tandem mass spectrometry (UPLC-MS/MS). As shown in Table 3, LFIA detected 3 negative samples (samples 2, 3, and 6) and 7 positive samples (samples 1, 4, 5, 7, 8, 9, and 10). Meanwhile, the detection results of UPLC-MS/MS show that the concentrations of CAP in samples 2, 3, and 6 are lower than the vLOD of LFIA in rice (0.04 mg/kg), and those in samples 1, 4, 5, 7, 8, 9, and 10 are more than 0.04 mg/kg. Excellent concordance was observed between the rAb-based LFIA and UPLC-MS/MS results, validating the accuracy and reliability of the LFIA for CAP in rice.

**Table 3:** Detection results by LFIA and UPLC-MA/MS for brown rice samples (*n* = 3)

Sample No	UPLC-MS/MS (mg/kg)	LFIA	Sample No	UPLC-MS/MS (mg/kg)	LFIA
1	0.48		6	0.008	
2	0.017		7	0.12	
3	0.005		8	0.29	
4	0.23		9	0.33	
5	0.35		10	0.06	

Note: (+): positive; (±) weakly positive; (−): negative

## Discussion

This work highlights important advancements in the production of recombinant mAbs for immunoassays via a promising *Pichia pastoris* expression system. First, expression plasmids for full-length CAP rAb were constructed. These plasmids can be readily adapted for the expression of other mAbs via routine enzymatic digestion and ligation procedures. Subsequently, high-level expression of rAb (9.3 mg/L) was achieved through systematic optimization of culture conditions, indicating that the system can significantly reduce the production costs of antibodies for immunoassay applications. Finally, the rAb of CAP exhibited better sensitivity in LFIA and reliable performance in agricultural sample detection, demonstrating the practicality of rAb expressed in yeast. Although this LFIA method achieves satisfactory detection performance in most agricultural matrices (rice, apples, and grapes), its vLOD for sweet potato samples is higher than the corresponding statutory MRL, leaving room for further sensitivity optimization. Future work will focus on improving the insufficient LFIA detection capability for CAP in sweet potato matrices. Specifically, further optimization of the QuEChERS pretreatment method will be performed to mitigate matrix interference and reduce dilution folds, while high-signal nanolabels will be adopted to enhance the overall detection sensitivity of the assay [42, 43]. The different types of glycosylation in various cells are a major challenge for stable full-length rAb expression. Here, a simple glycosylation site prediction was used to determine if the mAb is suitable for expression in a yeast host. In our future work, glycoengineered *Pichia pastoris* will be used to address these issues effectively [44]. Another limitation of this work is that the thermal stability and room-temperature storage performance of the yeast-expressed antibody were not evaluated. Glycosylation patterns differ significantly between yeast and mammalian expression systems, and these differences may substantially affect antibody stability, which is a critical determinant for the field applicability of LFIA strips. This important issue will be systematically investigated in future studies.

## Materials and methods

### Reagents

CAP (99.0%), broflanilide (95.4%), tetraniliprole (96.7%), 5-bromo-1-(3-chloropyridin-2-yl)−1H-pyrazole-3-carboxamide (95.2%), flubendiamide (95.4%), cyclaniliprole (94.0%), and benzamide (96.5%) were purchased from the Shanghai Pesticide Research Institute Co., Ltd. (Shanghai, China). *Pichia pastoris* yeast X-33 (his4⁻ Mut⁺) was stored in the laboratory, and mAb 5C5B9 and coat antigen CAP-OVA were developed in our previous work [30]. The pCDNA3.1-mouse-IgG1 heavy/kappa plasmids were a gift from Prof. Zhu Guonian’s group [45], and pPICZα A was purchased from Invitrogen (Carlsbad, CA, USA). T4 DNA Ligase enzyme, Xba Ⅰ, Sac Ⅱ, Pme Ⅰ, EcoR Ⅰ restriction endonucleases, and calf intestinal alkaline phosphatase (CIP) were purchased from New England Biolabs, Inc. (MA, USA). The seamless cloning kit was purchased from Wuhan Aibotech Biotechnology Co., Ltd. (Wuhan, China). The primers were synthesized by Shanghai Sangon Biotech. (Shanghai, China) and are shown in Table S1. Goat anti-mouse IgG was purchased from Boster (Pleasanton, CA, USA). Nitrocellulose (NC) membranes were purchased from Millipore (Boston, MA, USA). The sample pad (glass fiber, 8976), absorbent pad (Jn8301), polyvinyl chloride plate (PVC, Jn8100) plate and absorbent pad (H-5076) were purchased from Shanghai Jening Biotechnology Co., LTD (Shanghai, China). All organic reagents were of analytical purity, and biological reagents were prepared in double distilled water.

### Construction of expression plasmids

Total RNA was extracted from the hybridoma cell line 5C5B9 using RNAiso Plus reagent (Takara, Beijing, China) in accordance with the manufacturer’s standard protocols. The obtained total RNA was then reverse-transcribed into complementary DNA (cDNA) via PrimeScript™ II Reverse Transcriptase (Takara, Beijing, China). Subsequently, VH and VL were amplified by polymerase chain reaction (PCR) with the universal primers listed in Table S1.

CH and CL gene fragments were amplified from pCDNA3.1-mouse-IgG1 heavy and pCDNA3.1-mouse-IgG1 kappa plasmids using specific primers harboring Sac II and Xba I restriction enzyme sites. Two modified pPICZα A vectors carrying BleoR and NeoR resistance genes were separately generated via Gibson assembly, which were designed for the extracellular expression of antibody heavy chains and kappa light chains, respectively. Both the reconstructed pPICZα A vectors and amplified constant region gene fragments were subjected to double digestion with Sac II and Xba I restriction endonucleases. After digestion, the linearized vector backbones and target gene fragments were ligated. The recombinant plasmids were chemically transformed into competent *Escherichia coli* DH5α cells, and bacterial cells were spread onto Luria–Bertani (LB) solid plates supplemented with 50 μg/mL Zeocin or 200 μg/mL G418, followed by overnight incubation at 37 °C. Individual monoclonal colonies were randomly picked and validated via Sanger sequencing. The sequence-verified correct recombinant plasmids were designated pPICZα A-heavy and pPICZα A-kappa, respectively.

Subsequently, VH and VL gene fragments were separately inserted into the pre-constructed pPICZα A-heavy and pPICZα A-kappa expression vectors via Gibson assembly. Briefly, VH and VL target fragments were amplified by PCR using primers containing homologous recombination arms. The two backbone vectors were linearized via double digestion with EcoR Ⅰ and Sac II restriction endonucleases. The purified VH and VL fragments were individually inserted into corresponding linearized vectors using a seamless cloning kit (Aibotech, Wuhan, China). The resulting recombinant plasmids were used for chemical transformation of competent *Escherichia coli* DH5α cells. Positive clones were sent to Sangon Biotech (Nanjing, China) for Sanger sequencing verification. Finally, sequence-confirmed recombinant plasmids were designated pPICZα A-CAP-heavy and pPICZα A-CAP-kappa, respectively.

### Development of recombinant Pichia pastoris expression strains

Yeast competent cells were prepared via the lithium acetate method as previously described [46]. Briefly, wild-type *Pichia pastoris* X-33 strains were resuscitated by streaking on fresh yeast extract-peptone-dextrose (YPD) agar plates. The YPD medium consisted of 1% yeast extract, 2% peptone, 2% glucose and 2% agar, and the plates were incubated at 30 °C for 48 h. Single monoclonal yeast colonies were inoculated into 2 mL of liquid YPD medium in sterile 10 mL centrifuge tubes, followed by shaking incubation at 30 °C and 250 rpm for 12 h. The seed cultures were further transferred into 250 mL shake flasks supplemented with 50 mL YPD medium and cultivated under the same temperature and shaking speed until the optical density at 600 nm (OD_600 nm_) reached 1.3. The yeast cultures were harvested by centrifugation at 3000 g for 3 min, and the supernatants were discarded. Cell pellets were resuspended in 7 mL of 0.1 M lithium acetate solution mixed with 70 μL of 1 M DTT solution through gentle pipetting. The cell suspension was incubated at 30 °C with shaking at 110 rpm for 30 min. After another round of centrifugation (3000 × *g*, 3 min), the supernatant was removed, and the cell pellets were rinsed twice with 7 mL of sterile deionized water. Finally, cell precipitates were resuspended in 1 mL of pre-chilled 1 M sorbitol solution by vortexing to obtain yeast competent cells. Notably, the prepared competent cells were adopted immediately without long-term storage, given the rapid decline in transformation efficiency after preservation.

The constructed heavy chain expression plasmid pPICZα A-CAP-heavy was transformed into *Pichia. pastoris* X-33 competent cells first. Prior to electroporation, the recombinant plasmid was linearized via single digestion with Pme Ⅰ restriction endonuclease, followed by dephosphorylation treatment using CIP. For electroporation transformation, 50 μL of yeast competent cells were mixed with 5 μg of linearized plasmid in a 0.2 cm electroporation cuvette. Electroporation was performed on a Bio-Rad electroporator (California, USA) with optimized parameters of 1.5 kV voltage and 5 ms pulse duration. Immediately after electric shock, 950 μL of ice-cold 1 M sorbitol solution was added to the cuvette for gentle mixing. The mixture was transferred into a sterile 10 mL centrifuge tube and statically incubated at 30 °C for 1 h for cell recovery. Afterwards, the bacterial suspension was spread evenly onto YPD agar plates supplemented with 100 μg/mL Zeocin and cultured at 30 °C for 72 h to screen positive transformants. Single yeast clones were picked and cultured in 3 mL of liquid YPD medium for 12 h. Genomic DNA was extracted from cultured yeast cells using an EZNA Yeast DNA Kit (Omega, Doraville, USA). PCR amplification and subsequent Sanger sequencing were conducted using extracted genomic DNA as a template to verify the successful integration of the exogenous VH gene into *Pichia. pastoris* genome. Yeast strains carrying sequence-verified correct VH fragments were re-prepared into competent cells following the identical lithium acetate protocol mentioned above. Subsequently, the light chain plasmid pPICZα A-CAP-kappa was transformed into the above recombinant yeast competent cells. The cell recovery and positive screening procedures remained consistent with those described above, except that 200 μg/mL G418 was used for antibiotic selection instead of Zeocin.

### Expression of rAb

The verified recombinant yeast strains were streaked on YPD agar plates containing both 100 μg/mL Zeocin and 200 μg/mL G418, followed by incubation at 30 °C for 72 h. Single positive monoclonal colonies were inoculated into centrifuge tubes with 3 mL liquid YPD medium supplemented with the same dual antibiotics and cultivated at 30 °C and 250 rpm for 12 h to prepare seed cultures. The seed cultures were then transferred into 1 L shake flasks containing 200 mL buffered glycerol-complex medium (BMGY) for expanded cultivation. The strains were cultured at 30 °C and 250 rpm until the OD_600 nm_ reached 2. Subsequently, yeast cells were harvested via centrifugation at 3000 × *g* for 5 min, and the supernatants were completely discarded. Cell pellets were resuspended in buffered methanol-complex medium (BMMY) to adjust the OD_600 nm_ to 1.0 for methanol-induced antibody expression. The cell suspension was transferred to sterile new shake flasks, and the flask mouths were covered with double-layer sterile cheesecloth to ensure sufficient aerobic ventilation during fermentation. The cultures were incubated continuously at 30 °C and 250 rpm, and anhydrous methanol was supplemented every 24 h to maintain a final concentration of 0.5% (v/v) for persistent induction. After 120 h of continuous induction, the fermentation broth was centrifuged at 4000 × *g* for 10 min. The collected supernatant was purified using Protein A affinity chromatography columns (GenScript, Nanjing, China) to harvest purified recombinant antibodies (rAbs).

### Characterization of the rAb

Sodium dodecyl sulfate–polyacrylamide gel electrophoresis (SDS-PAGE) was performed to characterize the molecular weight and purity of the prepared full-length rAbs. A checkerboard titration assay was carried out to optimize the working concentrations of coated antigen and purified rAb, as described previously [28]. Based on the optimized reaction parameters, an ic-ELISA was further implemented to evaluate the sensitivity of the prepared recombinant antibody [29]. The cross-reactivity (CR) of rAbs was investigated using ic-ELISA to detect analogs of CAP. The CR values were calculated as follows:

CR (%) = (IC_50_ of CAP/IC_50_ of analog) × 100.

### Preparation of AuNPs and AuNP-rAbs

Gold nanoparticles (AuNPs) were synthesized via the classic sodium citrate reduction method as previously reported [47]. Briefly, 100 mL of 0.01% (w/w) chloroauric acid (HAuCl₄) aqueous solution was heated to a rolling boil and maintained for 2 min. Subsequently, 1 mL of 1% sodium citrate solution was quickly injected under continuous vigorous magnetic stirring. Once the solution turned wine red (characteristic color of mature AuNPs), the heating process was sustained for another 5 min. The resultant AuNP colloidal solution was then cooled naturally to room temperature.

For the preparation of AuNP-rAb conjugates, the pH value of the freshly prepared AuNP colloidal solution was adjusted to 8.2 using 0.1 M potassium carbonate solution. Afterwards, purified rAbs were added to the AuNP solution at a final concentration of 20 μg/mL, followed by 30 min of incubation in the dark at 25 °C to complete antibody immobilization. Unoccupied nonspecific binding sites on AuNP surfaces were further blocked with 1% bovine serum albumin (BSA, Sigma-Aldrich, MO, USA) for 30 min at 25 °C. The mixture was centrifuged at 8000 × *g* for 15 min, and the supernatant was discarded. Finally, the collected pellet was gently resuspended in a one-tenth volume of fresh 0.01 mol/L sodium borate buffer supplemented with 3% sucrose and 2% BSA for subsequent use.

### Assembly and procedure of LFIA

The conjugate pad was sprayed with 0.01 M sodium borate buffer containing 4% BSA at a spray volume of 5 μL/cm^2^, followed by drying in a vacuum drying oven at 37 °C for 30 min. Subsequently, AuNP-rAb conjugates were sprayed onto the pre-treated conjugate pad at an identical volume, and the pad was dried under the same temperature conditions for another 30 min. An XYZ-3000 dispensing platform (Bio-Dot, Irvine, CA, USA) was adopted to dispense two capture lines on the nitrocellulose (NC) membrane at a constant speed of 1 μL/cm: goat anti-mouse IgG (0.125 mg/mL) for the C line and coating antigen (1.25 mg/mL) for the T line. The functionalized NC membrane was then baked at 37 °C for 2 h for thorough immobilization. As illustrated in Fig. 4A, the complete LFIA strip consisted of five sequentially assembled components: sample pad, conjugate pad, NC membrane, absorbent pad and PVC bottom pad. All assembled strips were cut into 4 mm-wide individual strips and preserved under dry ambient conditions for subsequent detection.

For standard LFIA detection procedures, 100 μL of sample solution was loaded onto the sample pad, and detection results were visually recorded after a 10-min capillary flow reaction (Fig. 4B). The competitive detection mechanism was described as follows. In samples containing target analytes, analytes first bound specifically to AuNP-rAb conjugates via capillary flow when migrating to the conjugate pad. The formed analyte-AuNP-rAb complexes failed to bind with pre-coated antigens on the T line but could still be captured by goat anti-mouse IgG antibodies immobilized on the C line. Accordingly, the T line showed a faded red color or even complete color disappearance, while the C line maintained a distinct bright red color. In contrast, in analyte-free samples, unbound AuNP-rAb conjugates were captured by coating antigens on the T line, generating an obvious bright red T line. In summary, the detection criteria were defined clearly: a positive result was determined when the T line color was lighter than the C line or completely colorless; a negative result was confirmed when the T line color was comparable to or darker than the C line; any strips without visible red C lines were judged as invalid regardless of T line signals (Fig. 4B). The vLOD was defined as the minimum analyte concentration that could generate a valid positive LFIA result.

### Detection of spiked samples

Rice, apple, sweet potato and grape samples were selected to verify the practical detection performance of the developed LFIA method. All fresh food samples were chopped into small fragments and fully homogenized in advance. Briefly, 10 g of homogenized sample was accurately weighed and transferred into individual 50 mL centrifuge tubes. Subsequently, CAP standard working solution was added to different sample matrices to obtain spiked samples with concentrations of 0, 0.01, 0.02, 0.04 and 0.08 mg/kg. For pesticide extraction, 20 mL of extraction buffer containing 60% methanol was added to each spiked sample. The mixture was vigorously vortexed for 10 min, followed by 5 min of ultrasonic-assisted extraction. After centrifugation at 4000 × g for 5 min, the supernatants were appropriately diluted and used for LFIA detection.

### Verification by UPLC − MS/MS

UPLC-MS/MS (Waters, MA, USA) was adopted as the reference gold standard method to verify the accuracy and reliability of the established rAb-based LFIA. The correlation between the quantitative results obtained by LFIA and UPLC-MS/MS was analyzed to evaluate the practical performance of the strip assay. A total of ten rice samples fortified with unknown CAP concentrations were used for comparative detection. The detailed pretreatment procedures for UPLC-MS/MS detection are described as follows. In brief, 10 g of homogenized rice sample was transferred into a 50 mL centrifuge tube, followed by the addition of 20 mL mixed extraction solvent consisting of 0.1% formic acid aqueous solution and acetonitrile at a volume ratio of 1:1 (v/v). The mixture was vortexed vigorously for 15 min. Afterwards, 2.5 g of NaCl was added to facilitate liquid–liquid phase separation. After another 5 min of vortex oscillation, the mixture was centrifuged at 4000 × g for 5 min. A 3 mL aliquot of the upper organic supernatant was transferred into a 5 mL centrifuge tube preloaded with 75 mg C18 adsorbent and 150 mg N-propylethylenediamine (PSA) dispersive solid-phase extraction (dSPE) sorbents for impurity removal. The mixture was vortexed for 5 min and centrifuged again at 4000 × g for 5 min. Finally, 1 mL of purified supernatant was filtered through a 0.22 μm organic filter membrane prior to instrumental detection. All UPLC-MS/MS operating parameters were consistent with those reported in a previous study [30].

## Supplementary Information

Supplementary Material 1.

## Data Availability

All data generated or analyzed during this study are included in this published article and its supplementary information files.
