# Supplementary material for: Expression and immunoassay application of full-length recombinant antibody recognizing chlorantraniliprole using Pichia pastoris expression system
Source: Crop Health. 2026 Aug 24;4(1):23. doi: 10.1007/s44297-026-00085-4 (PMC13504142; doi:10.1007/s44297-026-00085-4)
Supplement: Supplementary file 1 [file 44297_2026_85_MOESM1_ESM.docx]

**Expression and immunoassay application of full-length recombinant antibody recognizing chlorantraniliprole using** ***Pichia pastoris* expression system.**

Lianrun Huang^1, 2^, Panpan Cui^1, 2^, Yang Zhang^1, 2^, Yanling Wu^1, 2^, Yuan Ding^1, 2^, Maojun Jin^3*^, Xiude Hua ^1, 2*,^

^1^College of Plant Protection, Nanjing Agricultural University, Nanjing , China

^2^State & Local Joint Engineering Research Center of Green Pesticide Invention and Application, Nanjing, China

^3^Institute of Quality Standards & Testing Technology for Agro-Products, Chinese Academy of Agricultural Sciences, Beijing, China

^*^Corresponding author. E-mail address: Maojun Jin: katonking@163.com; Xiude Hua: huaxiude@njau.edu.cn

**Table S1** The primers used in this study*.

| Name | Sequence 5’-3’ | PCR product |
| --- | --- | --- |
| V_H_-Forward1 | ATGAACTTYGGGYTSAGMTTGRTTT | VH |
| V_H_-Forward2 | ATGTACTTGGGACTGAGCTGTGTAT |  |
| V_H_-Forward3 | ATGAGAGTGCTGATTCTTTTGTG |  |
| V_H_-Forward4 | ATGGATTTTGGGCTGATTTTTTTTATTG |  |
| V_H_-Reverse | ACTAGTCGACATGGRATGGASCKKIRTCTTTMTCT |  |
| V_L_-Forward1 | ACTAGTCGACATGAAGTTGCCTGTTAGGCTGTTGGTGCT | VL |
| V_L_-Forward2 | ACTAGTCGACATGGATTTWCARGTGCAGATTWTCAGCTT |  |
| V_L_-Forward3 | ACTAGTCGACATGGTYCTYATVTCCTTGCTGTTCTGG |  |
| V_L_-Forward4 | ACTAGTCGACATGGTYCTYATVTTRCTGCTGCTATGG |  |
| V_L_-Reverse | ACTGGATGGTGGGAAGATGGA |  |
| CH-F | TCCCCGCGGGGCTAAAACGACACCCCCATCT | CH |
| CH-R | GACTCTAGAATCATTTACCAGGAGAG |  |
| CL-F | TCCCCGCGGGAGAGCAGATGCTGCACCAAC | CL |
| CL-R | GACTCTAGAATCAACACTCATTCCTG |  |
| VH-F | GAGAGGCTGAAGCTGAATTCGCTAAAACGACACCCCCA | VH with homologous arm |
| VH-R | TCCCTGGGCACCCCGCGGCTAATTTTCTTGTCCACCTTGGTG |  |
| VL-F | GAGAGGCTGAAGCTGAATTCATGACCCAGTCTCAAAAATTCAT | VL with homologous arm |
| VL-R | GCAGCATCTGCCCCGCGGCTCCGTTTTATTTCCAGCTTGG |  |

*: The primers used in this study were synthesized by Sangon Biotech (Shanghai) Co., Ltd. (Shanghai, China)

**Fig. S1** The construction process of the expression plasmids.

**Fig. S2** Agarose gel electrophoresis analysis of RNA extraction, target gene amplification and recombinant plasmid construction during recombinant antibody preparation. (A) Agarose gel electrophoresis of total RNA extracted from hybridoma cell line 5C5B9. Lane M: DNA marker; Lanes 1 and 2: two parallel total RNA samples from hybridoma 5C5B9. Arrows indicate the bands corresponding to 28S, 18S and 5S rRNA. (B) Amplification products of antibody variable region genes. Lane M: DNA marker; Lane VL: light chain variable fragment; Lane VH: heavy chain variable fragment. (C) Amplification products of antibody constant region genes. Lane M: DNA marker; Lanes 1 and 2: light chain constant region; Lanes 3 and 4: heavy chain constant region. (D) Identification of linearized empty vector pPICZαA. Lane M: DNA marker; Lane 1: circular pPICZαA plasmid; Lane 2: linearized pPICZαA plasmid. (E) Identification of recombinant plasmids pPICZαA-heavy and pPICZαA-kappa. Lane M: DNA marker; Lanes 1 and 2: amplified VL and VH fragments, respectively; Lanes 3 and 4: recombinant plasmids pPICZαA-heavy and pPICZαA-kappa. (F) Identification of final expression plasmids pPICZαA-CAP-heavy and pPICZαA-CAP-kappa. Lane M: DNA marker; Lane 1: pPICZαA-CAP-kappa plasmid; Lane 2: pPICZαA-CAP-heavy plasmid.

**Fig. S3** Results were verified by PCR for heavy (A) and light (B) chain positive transformants.

**Fig. S4** Results of the optimization of the working buffer solution. (A): Na+; (B): Tween-20; (C): pH; (D): Methanol (The concentrations of CAP from left to right were 2.5, 1.25, 0.63, 0.31, 0 ng/mL).

**Fig. S5** Detection results of CAP in different matrix effects by LFIA. (A) Brown rice; (B) Apple; (C) Sweet potato; (D) Grape; (E) Matrix free buffer solution.
